# Appendico‐Ileal Knotting: A Rare, Dangerous, and Often Overlooked Complication of Appendicitis: A Case Report

**DOI:** 10.1155/cris/6658226

**Published:** 2026-01-24

**Authors:** Temesgen Mamo Bisetegn, Yitayew Ewnetu Mohammed, Tekiy Markos Bedore, Yohannes Gizachew Achamyeleh, Tsedeke Tulicha Dorsisso

**Affiliations:** ^1^ Department of General Surgery, Worabe Comprehensive Specialized Hospital, Worabe, Ethiopia; ^2^ Department of Internal Medicine, Worabe Comprehensive Specialized Hospital, Worabe, Ethiopia; ^3^ Department of Emergency and Critical Care, Worabe Comprehensive Specialized Hospital, Worabe, Ethiopia; ^4^ Department of Internal Medicine, Saint Barnabas Hospital, New York, USA, sbhny.org; ^5^ Department of General Surgery, Saint Paul Millennium Medical College, Addis Ababa, Ethiopia, sphmmc.edu.et

**Keywords:** appendicitis, appendico-ileal knotting, case report, laparotomy, small bowel obstruction

## Abstract

Appendicitis and small bowel obstruction (SBO) are common causes of acute abdomen encountered in clinical practice. However, appendicitis as a cause of SBO is very rare and often overlooked, commonly resulting in delayed diagnosis and management. Appendico‐ileal knotting, a rare but dangerous way appendicitis could lead to mechanical SBO, occurs when an inflamed appendix forms a ring‐like structure through which a small bowel loop herniates, resulting in closed‐loop obstruction that could be complicated by small bowel strangulation and gangrene if not identified and intervened early. Preoperative diagnosis of appendico‐ileal knotting is very challenging, with most cases diagnosed intraoperatively. We present a case of a 35‐year‐old female who presented with crampy abdominal pain of 2 days duration associated with vomiting, abdominal distension, and constipation. Blood pressure was unrecordable, and abdominal examination was positive for diffuse guarding, rigidity, and rebound tenderness. A plain abdominal X‐ray revealed multiple centrally located air‐fluid levels, after which she was diagnosed with SBO and taken to the operating room for exploratory laparotomy. With the intraoperative finding of appendico‐ileal knotting complicated by distal ileal gangrene, she was managed with appendectomy, ileal resection, and end ileostomy. Appendico‐ileal knotting is a very rare condition with a limited number of case reports in the existing literature. This case report aims to contribute to a better understanding of this condition and emphasize the significance of early identification and intervention in reducing the substantial risk of morbidity and mortality associated with the condition if not managed timely.

## 1. Introduction

Small bowel obstruction (SBO) is the most common surgical disorder of the small intestine and one of the most common surgical emergencies encountered in clinical practice. Effective management of SBO requires a thorough understanding of the pathophysiology and timely identification of its cause. Worldwide intraperitoneal adhesion is the most common cause of mechanical SBO, followed by hernia and tumor [1]. In Ethiopia, the most common cause of SBO is volvulus. The second common cause of SBO is intussusception, followed by adhesion [2].

Appendico‐ileal knotting, also known as an appendicular tourniquet or appendiceal tie syndrome, is a very rare cause of mechanical SBO in which an inflamed appendix forms a ring‐like structure through which a small bowel loop herniates, resulting in closed‐loop obstruction. The small bowel herniation can occur with or without strangulation. Early identification and intervention could prevent strangulation, which otherwise could result in small bowel gangrene [3, 4]. Although the first documented case of appendico‐ileal knotting as a cause of mechanical SBO dates back to 1901, it is a very rare condition with only a few reported cases since then [5].

Preoperative diagnosis of SBO due to appendico‐ileal knotting is very challenging. Most patients usually present with features of acute SBO that cloud the clinical picture of acute appendicitis. In addition, limitations of the available imaging modalities and paucity of clinical experience on this rare condition contribute to the preoperative diagnostic difficulty. Therefore, a conclusive diagnosis is made intraoperatively. Surgical management may involve simple appendectomy, ileocecal and segmental resection, or right‐sided hemicolectomy depending on the severity and extent of bowel loop involvement intraoperatively [4].

Here, we report an interesting case of a 35‐year‐old female who presented with features of SBO, which intraoperatively was found to be due to appendico‐ileal knotting resulting in closed‐loop obstruction with distal ileal gangrene managed surgically with appendectomy, nonviable ileum resection, and end ileostomy. Ileostomy reversal was done after 4 months. This case report aims to be a valuable addition to the limited existing literature and contribute to a better understanding of this very rare cause of SBO.

## 2. Case Presentation

A 35‐year‐old female patient presented to the emergency department with a complaint of crampy abdominal pain of 2 days’ duration initially over the periumbilical region, which progressed to involve the whole abdomen. She also experienced nausea and frequent episodes of vomiting ingested matter that progressively became bilious. The patient also reported abdominal distention and failure to pass feces and flatus. Otherwise, she denied any previous history of surgery, trauma to the abdomen, chronic medical illness, or history of similar illness.

**Figure 1:**
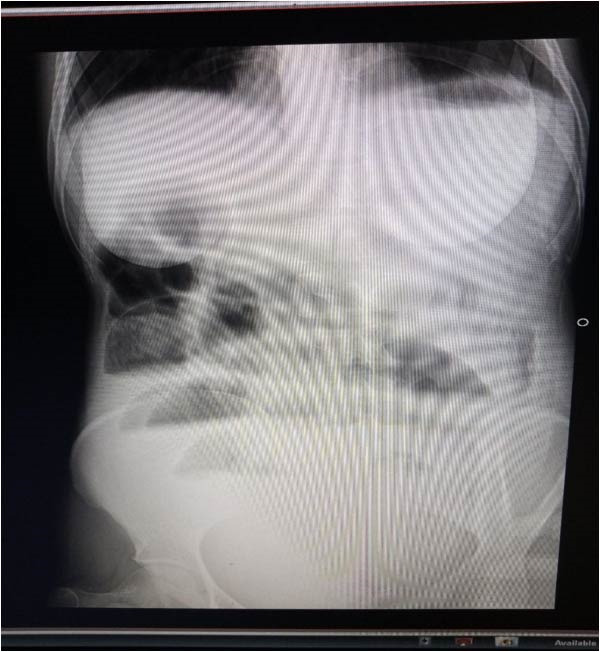
Preoperative plain erect abdominal X‐ray: note multiple centrally located air‐fluid levels with absent rectal shadow.

On physical examination, the patient was acutely sick‐looking with unrecordable blood pressure, a feeble radial pulse of 118 beats/minute, a respiratory rate of 26 breaths/minute, a temperature of 36.5°C, and oxygen saturation of 90% on room air. Abdominal examination revealed a distended abdomen with visible peristalsis, diffuse guarding, and rigidity with positive direct and rebound tenderness. A digital rectal examination revealed a normal anal tone with an empty rectum. Laboratory investigations revealed WBC = 18,000/μl (neutrophil = 87%, lymphocytes = 11%), Hgb = 12.9 g/dL, HCT = 41%, serum creatinine = 2.23 mg/dL, and BUN of 83 mg/dL. A plain erect abdominal X‐ray shows multiple centrally located air‐fluid levels with an absent rectal shadow (Figure 1).

With a provisional preoperative assessment of SBO secondary to primary small bowel volvulus complicated by septic shock and prerenal acute kidney injury (AKI), an IV line was secured and initial resuscitation with crystalloid was started. A nasogastric tube and urinary Foley catheter were inserted. Ceftriaxone 1 gm IV BID and metronidazole 500 mg IV TID were started. With initial resuscitation of 1500 mL of normal saline over 1 h, a recordable BP of 90/55 mmHg and adequate urine output of 80 mL over the first hour were achieved, and the patient was immediately taken to the operating room where exploratory laparotomy was done through a midline vertical incision.

**Figure 2:**
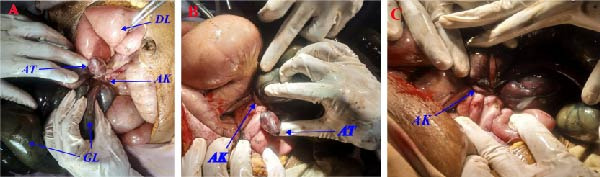
(A–C): Intra‐operative findings: note the appendicular knot (AK), gangrenous distal ileal loop (GL), dilated small bowel loop (DL) proximal to the obstruction, and the swollen and gangrenous tip of the inflamed appendix (AT).

**Figure 3:**
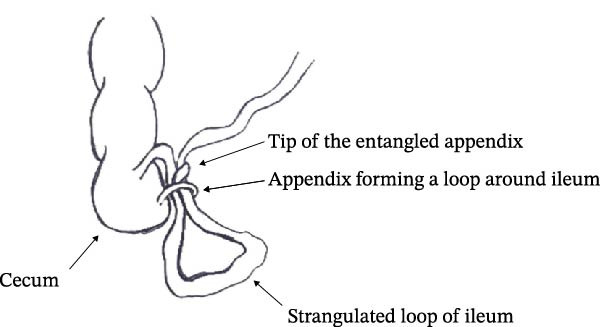
Pictorial illustration of the appendico‐ileal knot observed in the presented case.

Intraoperatively, there was around 1500 mL of hemorrhagic fluid in the general peritoneum. The appendix was identified as being entangled with the distal loop of the ileum and its mesentery. The inflamed and elongated appendix was found wrapped around the distal ileum, forming a knot and encircling two points of the distal ileum (at around 10 cm and 160 cm proximal to the ileocecal valve), causing a closed‐loop obstruction. The entangled part of the distal ileum, measuring about 150 cm long, and the whole length of the appendix were gangrenous. The jejunum and part of the ileum proximal to the obstruction were distended and viable with a pink appearance, visible peristalsis, and adjacent mesenteric vessel pulsation (Figures 2 and 3).

We divided the appendix at the shaft, and en bloc (without untwisting the entanglement) resection of the gangrenous ileum was done. Retrograde appendectomy was completed by dividing the appendix from the cecal base. The remaining bowel was mopped with a warm saline‐soaked pack, and the abdomen was thoroughly lavaged with warm saline. Due to intraoperative hemodynamic instability, end‐to‐end anastomosis was deferred, and end ileostomy was done. Finally, the abdomen was closed layer by layer with no drainage tube left. Immediately after the procedure, the patient was transferred to the ICU for postoperative follow‐up and kept there for 2 days before being transferred to the ward on the third postoperative day. She began taking a fluid diet on the second postoperative day after an active bowel movement sound was heard.

**Figure 4:**
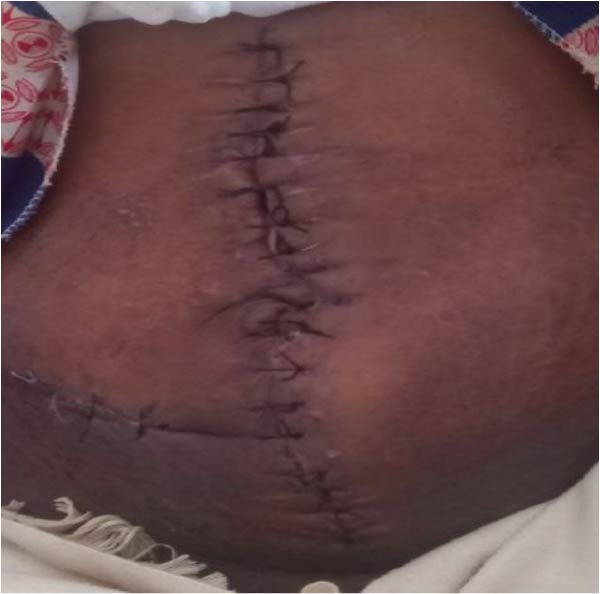
4^th^ postoperative day after ileostomy reversal.

After 15 days of stay in the hospital for completion of antibiotics, the patient was discharged on the 14^th^ postoperative day with stable vital signs and a functional ileostomy. On follow‐up, she did not have any complaints except wound site pain. On the 4^th^ postoperative month, she was admitted to the surgical ward with the diagnosis of functional ileostomy for ileostomy reversal. Subsequently, the ileostomy reversal was done with ileocolic anastomosis (Figure 4). Finally, she was discharged on the 7^th^ postoperative day with stable vital signs and no postoperative complications.

## 3. Discussion

Appendicitis and SBO are common surgical emergencies in clinical practice; however, they rarely occur in the same presentation [6]. The association between acute appendicitis and mechanical SBO was first described in 1901 by Lucius Hotchkiss, who reported 3 cases of mechanical SBO due to appendicitis successfully managed with surgery [5].

Appendicitis can induce SBO through various mechanisms. Ahmed et al. in 2019 classified SBO secondary to appendicitis pathologically into two subtypes: functional and mechanical. Functional SBO may result from paralytic ileus or, very rarely, due to mesenteric ischemia. Paralytic (adynamic) SBO, the most common form of SBO due to appendicitis, occurs when appendicular inflammation spreads to the adjacent bowel wall [7, 8]. Mechanical SBO due to appendicitis could also occur in various ways. The most common mechanism of mechanical obstruction is kinking, compression, or traction of the small bowel by an inflammatory process in the appendix, such as appendicular mass or abscess [7–9].

Appendico‐ileal knotting is a less common mechanism of mechanical SBO in the setting of appendicitis. However, it is a very dangerous condition where the ensuing SBO is of the closed‐loop type, which may rapidly result in small bowel strangulation, gangrene, and perforation. An appendico‐ileal knot can occur in two ways: (a) an elongated inflamed appendix wrapping around the base of a bowel loop, and (b) an inflamed appendix adhering to the cecum, small intestine, or posterior peritoneum, and a part of the bowel herniating through the gap [8]. Appendiceal tie syndrome, appendiceal band syndrome, and appendiceal tourniquet are some of the other terms used in literature to describe this condition [3, 7, 10–13]. In our case, the appendico‐ileal knotting was caused by the wrapping around of an elongated inflamed appendix over the loop of distal ileum and its mesentery, causing distal ileal strangulation and gangrene.

Preoperative diagnosis of appendico‐ileal knotting is very challenging, as the clinical picture of intestinal obstruction dominates in most cases and clinical examination is usually not typical for appendicitis. Right iliac tenderness is only evident in a few cases and is usually attributed to small bowel ischemia [7]. The rarity of this condition also makes diagnosis further challenging. A computed tomography (CT) scan of the abdomen can suggest the diagnosis preoperatively in the early inflammation phase. However, after the resolution of appendicitis, its role is very limited [14]. Although a CT scan may help diagnose and localize the source of SBO, it is not necessary, and surgery should not be delayed for a scan [6]. Moreover, while history, physical examination, imaging findings, and a high index of suspicion may help in diagnosis, a conclusive diagnosis of appendico‐ileal knotting is made intraoperatively [4].

In our patient, a preoperative diagnosis of SBO was suspected based on clinical presentation and confirmed using a plain erect abdominal X‐ray. The definitive diagnosis of appendico‐ileal knotting was established intraoperatively. As the patient presented with unstable hemodynamic status, an abdominal CT scan was deferred, and she was immediately taken to the operating room for exploratory laparotomy.

The surgical management of appendico‐ileal knotting is tailored to the severity and extent of bowel loop involvement. It encompasses a range of procedures, from simple appendectomy to segmental and ileocecal resection to a right‐sided hemicolectomy [4]. With the expanding role of laparoscopy, recent experiences have demonstrated that a obtaining diagnosis and subsequent management can be done safely laparoscopically. This provides the advantage of a shorter hospital stay, faster return of bowel function, fewer wound infections, early recovery, and reduced incidence of subsequent adhesion SBO [6].

## 4. Conclusion

Appendico‐ileal knotting is a very rare cause of mechanical SBO. Surgeons and emergency physicians who deal with patients with an acute abdomen on a daily basis should familiarize themselves with this rare condition and understand the significance of early consideration of appendico‐ileal knotting in the appropriate clinical setting for timely intervention to prevent the substantial risk of morbidity and mortality associated with the condition if not managed timely.

## Acknowledgments

The authors would like to acknowledge the team of health care professionals involved in the management of the case.

## Funding

No funding was received by the authors for the preparation of this case report.

## Consent

Consent was taken from the patient before writing this manuscript.

## Conflicts of Interest

The authors declare no conflicts of interest.

## Data Availability

The data that support the findings of this study are available from the corresponding author upon reasonable request.
